# Clinical comparison of the BIOFIRE Joint Infection Panel using synovial fluid and tissue biopsy samples

**DOI:** 10.1128/spectrum.03402-25

**Published:** 2026-02-20

**Authors:** Llanos Salar-Vidal, Jaime Esteban, Frédéric Laurent, Lelia Abad, Arin Putnam, Corrin Graue, Bart Kensinger

**Affiliations:** 1 IIS-Fundación Jiménez Díaz, UAM, Madrid, Spain; 2 CIBERINFEC-CIBER de Enfermedades Infecciosas, Madrid, Spain; 3 Hospices Civils de Lyon 26900, Lyon, France; 4 bioMérieux, Inc., Salt Lake City, Utah, USA; Children's National Hospital, George Washington University, Washington, DC, USA

**Keywords:** bone and joint infections, molecular biology, multiplex PCR, tissue biopsy

## Abstract

Bone and joint infections pose significant clinical challenges due to their complexity and the associated high morbidity. Traditional microbiological culture is the gold standard, but has several limitations, including slow time to results and low sensitivity (i.e., culture produces false negative results, especially in patients on antibiotics before sample collection). In recent years, molecular diagnostic techniques, such as the BIOFIRE Joint Infection (JI) Panel, have been developed to overcome these challenges. This study aims to evaluate the diagnostic yield of tissue biopsies (TBs) compared to synovial fluid (SF) specimens in detecting pathogens responsible for these infections using the BIOFIRE JI Panel. The study included 151 SF specimens and 324 paired TBs from 151 subjects with suspected septic arthritis or prosthetic joint infection. Results showed that the BIOFIRE JI Panel had a high diagnostic yield, detecting pathogens in 31.7% of SF specimens and 36.4% of TB specimens, with a high concordance (Kappa = 0.77) between SF BIOFIRE JI Panel and SF culture results, and even better concordance (Kappa = 0.80) between TB BIOFIRE JI Panel and TB culture results. Notably, the BIOFIRE JI Panel demonstrated a higher detection rate in polymicrobial infections, identifying additional microorganisms compared to culture. The study concluded that the BIOFIRE JI Panel improves the diagnostic yield compared to conventional culture, particularly in cases of polymicrobial infections, and that testing TBs can provide valuable information when SF samples yield negative or inconclusive results. These findings suggest that PCR testing on TBs could be a useful adjunct to improve the diagnosis and management of bone and joint infections, especially when a prompt result is required.

**IMPORTANCE:**

Bone and joint infections are difficult to diagnose and treat, often leading to prolonged illness and serious complications. Traditional culture methods, while widely used, can be slow and sometimes miss the true cause of infection, especially if patients are already receiving antibiotics. This study shows that a rapid molecular test, the BIOFIRE Joint Infection Panel, can improve the detection of bacteria in both joint fluid and tissue samples. Importantly, the test was able to identify more infections, including those caused by different species of bacteria, compared to conventional methods. These findings highlight the potential of this approach to provide faster and more accurate results, which could help clinicians choose the right treatment earlier. By supporting quicker and more reliable diagnoses, this method may ultimately improve outcomes for patients with complicated bone and joint infections.

## INTRODUCTION

Bone and joint infections, including septic arthritis, prosthetic joint infection (PJI), osteomyelitis, and spinal infections, are associated with high morbidity (1, 2). They are difficult to treat, often requiring surgery, prolonged antimicrobial therapy, and posing a risk of complications such as sepsis, joint destruction, chronic pain, and disability (2). This results in an increased economic burden on the healthcare system (3).

The diagnosis of this type of infection is complex, and effective management requires a multidisciplinary team. Etiological diagnosis through microbiological testing is crucial for treatment success. Although traditional microbiological culture remains the gold standard for etiological diagnosis, it presents several limitations, such as slow turnaround time and false negative results, especially in patients receiving antibiotics (2, 4–6).

In this context, to enhance microbiological diagnosis, various molecular biology techniques have been developed in recent years to overcome the limitations of traditional culture methods (7). The BIOFIRE Joint Infection (JI) Panel (bioMérieux SA, BioFire Diagnostics, LLC) is a rapid multiplex-PCR diagnostic assay for the detection and identification of 31 microorganisms, as well as eight antimicrobial resistance genes commonly associated with septic arthritis and PJI (Table 1). The multicenter evaluation of the tool has shown significant value in terms of sensitivity (90.9%) and specificity (98.5%) in synovial fluid (henceforth SF) samples (8).

**TABLE 1:** List of microorganisms and antimicrobial resistance markers included in the BIOFIRE JI Panel

Gram-positive bacteria		
*Anaerococcus prevotii/vaginalis*	*Parvimonas micra*	*Streptococcus* spp.
*Clostridium perfringens*	*Peptoniphilus*	*Streptococcus agalactiae*
*Cutibacterium avidum/granulosum*	*Peptostreptococcus anaerobius*	*Streptococcus pneumoniae*
*Enterococcus faecalis*	*Staphylococcus aureus*	*Streptococcus pyogenes*
*Enterococcus faecium*	*Staphylococcus lugdunensis*	
*Finegoldia magna*		

Gram-negative bacteria		
*Bacteroides fragilis*	*Kingella kingae*	*Proteus* spp.
*Citrobacter*	*Klebsiella aerogenes*	*Pseudomonas aeruginosa*
*Enterobacter cloacae* complex	*Klebsiella pneumoniae* group	*Salmonella* spp.
*Escherichia coli*	*Morganella morganii*	*Serratia marcescens*
*Haemophilus influenzae*	*Neisseria gonorrhoeae*	

Yeast		
*Candida*		
*Candida albicans*		

Antimicrobial resistance genes		
CTX-M	*mecA/C* and MREJ (MRSA)	*vanA/B*
IMP	NDM	VIM
KPC	OXA-48-like	

Sometimes, obtaining a microbiological pathogen detection using SF is not possible due to the inability to collect the sample from the patient, or when the culture of the SF yields a negative result. In such cases, the analysis of tissue biopsies obtained under sterile conditions (henceforth TBs) with a PCR method might be helpful in providing a timely result.

In the present study, we evaluate the diagnostic yield of TB samples (a sample type not indicated for use in the manufacturer’s instructions for use [IFU]) compared to SF samples for the detection and identification of potential pathogens responsible for septic arthritis and PJI using the BIOFIRE JI Panel. Because of the large number of BIOFIRE JI Panel and culture results, we first summarize the results of the 42 subjects with monomicrobial detections (according to the BIOFIRE JI Panel). We then provide a more detailed examination of the 19 subjects with polymicrobial detections (according to the BIOFIRE JI Panel). An additional detailed examination of the 42 subjects with monomicrobial detections (according to the BIOFIRE JI Panel) is provided in.

## MATERIALS AND METHODS

### Clinical specimens

The study was conducted at two geographically distinct E.U. sites (Hospices Civils de Lyon, Lyon, France, and Hospital Universitario Fundación Jiménez Díaz, Madrid, Spain) over a period of approximately 1 year and 9 months (May 2018 to February 2020). Between May 2018 and August 2019, specimens were collected and immediately frozen for later testing. Between August 2019 and February 2020, specimens were collected and tested fresh; previously enrolled, frozen specimens were also thawed and immediately tested during this time. Residual SF and TBs from subjects of all ages were enrolled. Specimens were neat SF (i.e., have not been diluted in any type of transport media and were not collected on a swab, etc.) paired with at least one, and up to four, tissue specimens from the same subject. All TBs were homogenized in molecular-grade water or PBS before testing. All SF and TB were collected in sterile, rigid, plastic containers. Additionally, specimens were left over from Standard of Care (SOC) culture testing for suspected bone or joint infection (as defined by a physician-ordered culture performed on the SF specimen), placed in a refrigerator (~4°C) as soon as possible after collection, and stored for less than or equal to seven days before enrollment. Sufficient SF volume was required to be available for use in the study (900 μL for subjects 18 years of age and over; 600 μL for subjects 17 years of age and under). All specimens were from subjects that had not previously been enrolled in the study. A waiver of the requirement for informed consent was obtained from the Ethics Committees at both study sites for the use of residual SF and TB specimens, and for the collection of information from the medical records of the enrolled subjects, including clinical and demographic data. Clinical and demographic data included date of specimen collection, subject age range, subject sex, the location of the infected joint, the presence or absence of a prosthesis, the results of the clinician-ordered SOC culture performed on the SF and TB specimens, and phenotypic antimicrobial susceptibility testing of clinical isolates.

### BIOFIRE JI Panel testing

This study was conducted with an investigational-use-only version of the BIOFIRE JI Panel that is identical to the commercial (i.e., FDA-cleared, CE-marked) *in vitro* diagnostic version. All specimen handling occurred in a biosafety cabinet with operators wearing appropriate personal protective equipment, preparing one specimen at a time, and cleaning between specimens, all according to the manufacturer’s IFU (9). Approximately 200 μL of each specimen was subject to BIOFIRE JI Panel testing. The BIOFIRE JI Panel consists of automated nucleic acid extraction, reverse transcription, nucleic acid amplification, and automated results analysis in approximately 1 h per run (i.e., per specimen). If either internal control fails, the software automatically provides a result of “Invalid” for all panel analytes. Microorganisms and AMR genes are reported qualitatively as “Detected” or “Not Detected.” AMR genes are only reported if one or more applicable bacteria (i.e., potential carriers of the AMR gene) are also “Detected”; if no applicable bacteria are “Detected,” the AMR gene results are reported as “N/A” (not applicable).

### Standard-of-care culture comparator

Both study sites followed their own standard, validated procedures to determine SOC culture results, independent of the study, and culture was always performed from fresh specimens. Results for all SF and TB specimens were obtained from the chart review of subjects’ medical information.

### Statistical analysis

Kappa’s coefficient was used to determine the agreement between the diagnostic techniques. Statistical analysis was performed with the R statistical language version 4.2.1.

### Composite results

For the analyses presented below, because of the unequal number of TB samples collected and tested for each subject, several analyses refer to a composite result for the TB samples for both the BIOFIRE JI Panel result and the culture result. The composite results were calculated such that a positive result from any individual test resulted in a composite positive; all individual tests being negative resulted in a composite negative.

## RESULTS

During the study period, 151 SF specimens (i.e., one from each subject) and 324 TBs were collected (between 1 and 4 TBs from each subject). Specifically, exactly one TB was collected from 57 subjects, exactly two TBs were collected from 38 subjects, exactly three TBs were collected from 33 subjects, and exactly four TBs were collected from 23 subjects. Overall, the ratio of SF to TB was 1:2.14. Of the 151 subjects, 110 subjects’ specimens were tested frozen, and 41 subjects’ specimens were tested fresh on the BIOFIRE JI Panel; for the frozen specimens, the average number of days a specimen remained frozen (before thawing and immediately testing) was 494 (SD = 71) days. Most of the subjects were age 65 or older (68.9%), with 52.3% being male. The majority of subjects’ samples were obtained from subjects with prosthetic joints (132/151; 87.4%). For one subject, it was not possible to determine from the medical records whether the joint contained a prosthesis or not. The remaining subjects’ samples were obtained from a native joint (18/151; 11.9%). The principal source was the knee (79/151; 52.3%), followed by hip (63/151; 41.7%), and other (6/151; 4.0%). The source was unknown in three cases. The BIOFIRE JI Panel SF test for each subject gave at least one positive organism result in 48 (31.7%) subjects, with nine of these subjects showing multiple organism results (Table 2 and Table 5). The BIOFIRE JI Panel composite TB tests gave at least one positive organism result in 55 (36.4%) subjects, with 15 of these subjects showing multiple organism results (Table 2 and Table 5). In 87.5% (42/48) of subjects where the SF specimen tested positive by the BIOFIRE JI Panel, the microorganism(s) identified by the panel exactly matched the composite TB results on the BIOFIRE JI Panel.

**TABLE 2:** Summary of the 42 monomicrobial subjects

BIOFIRE JI Panel analyte	Monomicrobial detections (*n* = 42 subjects)			
	# of subjects with BIOFIRE JI Panel SF detections	# of subjects with culture SF detections^*a*^ (culture ID)	# of subjects with BIOFIRE JI Panel TB composite detections^*b*^	# of subjects with culture TB composite detections^*a*^^,*b*^ (culture ID)
*Enterococcus faecalis*	3	3 (*Enterococcus faecalis*)	3	3 (*Enterococcus faecalis*)
*Peptoniphilus*	1	1 (*Peptoniphilus assacharolyticus*)	1	1 (*Peptoniphilus assacharolyticus*)
*Staphylococcus aureus*	18	14 (*Staphylococcus aureus*)	20	15 (*Staphylococcus aureus*)
*Staphylococcus lugdunensis*	1	–^*c*^	–	–
*Streptococcus* spp.	3	1 (*Streptococcus dysgalactiae*) 1 (*Streptococcus gordonii*)	3	1 (*Streptococcus anginous*) 1 (*Streptococcus dysgalactiae*) 1 (*Streptococcus gordonii*)
*Streptococcus agalactiae*	1	1 (*Streptococcus agalactiae*)	1	1 (*Streptococcus agalactiae*)
*Enterobacter cloacae*	1	2 (*Enterobacter cloacae*)	3	2 (*Enterobacter cloacae*)
*Escherichia coli*	1	1 (*Escherichia coli*)	1	–
*Klebsiella pneumoniae* group	1	1 (*Klebsiella pneumoniae*)	1	–
*Proteus* spp.	1	1 (*Proteus mirabilis*)	2	1 (*Proteus mirabilis*)
*Pseudomonas aeruginosa*	1	1 (*Pseudomonas aeruginosa*)	–	–
*Serratia marcescens*	–	–	1	1 (*Serratia marcescens*)
*Candida albicans*	1	2 (*Candida albicans*)	1	1 (*Candida albicans*)

^*a*^ Culture results are not shown for microorganisms that the BIOFIRE JI Panel is not designed to detect.

^*b*^ For composite results, any positive result in any sample was considered a detected result for the subject.

^*c*^ “–” indicates that no subjects were identified in that category (i.e., zero subjects).

Overall, comparing the BIOFIRE JI Panel SF and the BIOFIRE JI Panel composite TB results, two subjects had additional organism detections by testing the SF (compared to the TBs), and six subjects had additional organism detections by testing all the TBs available for the subject (compared to the single SF result).

Anonymized individual results are provided in the Supplemental material.

### Monomicrobial subject BIOFIRE JI Panel comparison

Monomicrobial subjects are defined here as subjects for which the sum of all BIOFIRE JI Panel SF and TB results produced one or more detections, with all detections being for a single microorganism. Of the 42 monomicrobial subjects, 28 subjects had positive results on both SF and TB, five had a positive result only on SF, and nine had a positive result(s) only on TB (Table 2). Monomicrobial results are summarized in Table 2, showing the number of subjects with positive BIOFIRE JI Panel SF result, SF culture results, BIOFIRE JI Panel composite TB results, and composite TB culture results for each organism. Seven microorganisms (*Enterococcus faecalis, Peptoniphilus, Streptococcus* spp.*, Streptococcus agalactiae, Escherichia coli, Klebsiella pneumoniae* group*,* and *Candida albicans*) had the same number of subjects with BIOFIRE JI Panel detections on the SF and the composite TB result, two microorganisms (*S. lugdunensis* and *P. aeruginosa*) had more subjects (1 each) with BIOFIRE JI Panel detections by the SF result compared to the BIOFIRE JI Panel composite TB result, and four microorganisms (*Staphylococcus aureus*, *Enterobacter cloacae*, *Proteus* spp., and *Serratia marcescens*) had more subjects (2, 2, 1, and 1, respectively) with BIOFIRE JI Panel detections with the composite TB result compared to the BIOFIRE JI Panel SF result.

### Monomicrobial infections statistical analyses

Two analyses were performed on the data set containing monomicrobial subjects. First, concordance between the BIOFIRE JI Panel results when testing SF specimens and the BIOFIRE JI Panel results when testing TBs was assessed by calculating the Kappa coefficient. According to the interpretation of the kappa coefficient, we consider agreement as follows: κ < 0.2 is very poor, 0.2 ≤ κ < 0.4 is poor, 0.4 ≤ κ < 0.6 is moderate, 0.6 ≤ κ < 0.8 is good, and κ ≥ 0.8 is very good. Good concordance (kappa = 0.73) was detected between BIOFIRE JI Panel results from SF specimens and TBs (Table 3). Finally, concordance between the BIOFIRE JI Panel results when testing SF versus TB and culture results when testing the corresponding specimen type was assessed by calculating the kappa coefficient. Good concordance (kappa = 0.77) was detected between BIOFIRE JI Panel results from SF and culture, and for BIOFIRE JI Panel results from TB and culture (kappa = 0.80) (Table 4).

**TABLE 3:** Concordance between BIOFIRE JI Panel used on SF compared to BIOFIRE JI Panel used on TBs for the monomicrobial subjects

BIOFIRE JI Panel SF	Composite BIOFIRE JI Panel TB		Kappa	Accuracy (%)
	Negative	Positive		
Negative	90	9	0.73 (0.60–0.76)	89.4 (82.8–94.1)
Positive	5	28		

**TABLE 4:** Concordance between BIOFIRE JI Panel result when used on SF compared to SF culture, and the composite of the BIOFIRE JI Panel TB results compared to the composite TB culture for monomicrobial subjects

Tested	BIOFIRE JI Panel	Culture		Kappa	Accuracy (%)
		Negative	Positive		
Synovial fluid	Negative	95	4	0.77 (0.64–0.90)	91.7 (85.6–95.8)
	Positive	7	26		
Composite TB	Negative	94	1	0.80 (0.68–0.92)	91.7 (86.5–96.3)
	Positive	9	28		

### Polymicrobial subjects BIOFIRE JI Panel comparison

Polymicrobial subjects are defined here as subjects for which the sum of all BIOFIRE JI Panel SF and TB results produced two or more detections with a minimum of two microorganisms identified. All polymicrobial subjects’ test results are shown in Table 5. Of the 19 polymicrobial subjects, 14 subjects had positive result(s) on both SF and TB, one had a positive result only on SF, and four had positive result(s) only on TB. In total, 19 subjects had polymicrobial detections. In four subjects, additional microorganisms were detected by the BIOFIRE JI Panel in TB specimens when SF testing was negative. In another six subjects, only one microorganism was detected by the BIOFIRE JI Panel in SF, while additional microorganisms were detected in the TBs. Finally, in four subjects, the BIOFIRE JI Panel detected more microorganisms in the SF than the TBs.

**TABLE 5:** All results for the 19 polymicrobial subjects^*a*^

Subject	BIOFIRE JI Panel SF	Culture SF^*a*^	BIOFIRE JI Panel TB	Culture TB^*a*^	# of tissue biopsies
1	*Enterococcus faecalis*	*Enterococcus faecalis*	*Enterococcus faecalis* (3), *Staphylococcus aureus*	*Enterococcus faecalis* (3)	4
2	*Candida, Candida albicans*	*Candida albicans*	*Enterococcus faecalis, Proteus* spp., *Candida, Candida albicans*	Negative	1
3	*Enterococcus f*a*ecalis*, *Proteus* spp.	Negative	Negative	Negative	3
4	Negative	Negative	*Anaerococcus prevotii/vaginalis* (3), *Staphylococcus lugdunensis* (4)	*Cutibacterium avidum* (4), *Staphylococcus lugdunensis* (4)	4
5	*Anaerococcus prevotii/vaginalis, Finegoldia magna, Streptococcus* spp.	*Anaerococcus prevotii/vaginalis, Finegoldia magna, Streptococcus constellatus*	*Anaerococcus prevotii/vaginalis* (2), *Finegoldia magna* (2), *Staphylococcus aureus, Streptococcus* spp. (2)	*Finegoldia magna* (3), *Streptococcus constellatus* (4), *Proteus mirabilis*	4
6	*Staphylococcus lugdunensis, Proteus* spp.	Negative	*Staphylococcus lugdunensis* (2)	*Staphylococcus lugdunensis*	2
7	*Escherichia coli, Proteus* spp.	*Escherichia coli, Proteus mirabilis*	*Escherichia coli, Proteus* spp.	*Escherichia coli, Proteus mirabilis*	1
8	Negative	Negative	*Enterobacter cloacae* complex, *Klebsiella pneumonia*e group	Negative	1
9	*Staphylococcus aureus*	*Staphylococcus aureus*	*Staphylococcus aureus* (4), *Staphylococcus lugdunensis*	*Staphylococcus aureus* (4)	4
10	*Enterococcus faecalis, Finegoldia magna, Peptoniphilus, Peptostreptococcus anaerobius, Klebsiella pneumonia*e group, *Morganella morganii*, *Proteus* spp.	*Enterococcus faecalis, Klebsiella pneumoniae, Proteus mirabilis*	*Enterococcus faecalis* (2), *Peptoniphilus* (2), *Peptostreptococcus anaerobius* (2), *Morganella morganii* (2), *Proteus* spp. (3), *Pseudomonas aeruginosa* (2)	*Enterococcus faecalis* (3), *Klebsiella pneumoniae, Proteus mirabilis* (3)	3
11	Negative	Negative	*Enterococcus faecalis, Escherichia coli* (2)	*Escherichia coli* (2)	2
12	Negative	Negative	*Parvimonas micra, Staphylococcus aureus, Streptococcus* spp.	Negative	1
13	*Enterococcus faecalis, Staphylococcus aureus*	*Enterococcus faecalis, Staphylococcus aureus*	*Enterococcus faecalis* (2), *Staphylococcus aureus* (3)	*Enterococcus faecalis* (3), *Staphylococcus aureus* (3)	3
14	*Klebsiella pneumonia*e group, *Morganella morganii*	Negative	*Enterococcus faecalis* (2), *Enterococcus faecium, Finegoldia magna, Klebsiella pneumonia*e group, *Morganella morganii* (2)	*Enterococcus faecalis, Klebsiella pneumoniae, Morganella morganii* (2)	4
15	*Staphylococcus aureus, Escherichia coli*	*Escherichia coli*	*Escherichia coli* (3)	*Escherichia coli* (3)	3
16	*Escherichia coli*	*Escherichia coli*	*Staphylococcus aureus, Staphylococcus lugdunensis, Escherichia coli* (3)	*Escherichia coli* (3)	3
17	*Streptococcus* spp.	*Streptococcus anginosus*	*Staphylococcus aureus, Streptococcus* spp. (2)	*Streptococcus anginosus*	3
18	*Morganella morganii*	*Morganella morganii*	*Streptococcus* spp., *Morganella morganii* (4)	*Morganella morganii* (4)	4
19	*Staphylococcus aureus, Serratia marcescens*	*Staphylococcus aureus*	*Staphylococcus aureus*	*Staphylococcus aureus*	1

^*a*^ Culture results for microorganisms that the BIOFIRE JI Panel is not designed to detect are not shown.

Two subjects had BIOFIRE JI Panel composite TB results (*E. cloacae* complex, *K. pneumoniae* group/*Parvimonas micra*, *S. aureus*, *Streptococcus* spp.) where BIOFIRE JI Panel SF, SF culture, and TB culture were all negative. One subject had BIOFIRE JI Panel SF results (*E. faecalis*, *Proteus* spp.) where the BIOFIRE JI Panel composite TB, SF culture, and TB culture were all negative.

### BIOFIRE JI Panel and culture comparison

Comparing the monomicrobial subjects’ BIOFIRE JI Panel SF results and the SF culture results, 34 of 42 subjects’ results were concordant; 27 were positive, and 7 were negative (Table 2). Regarding the discordant results, six additional results were obtained with the BIOFIRE JI Panel SF: four *S*. *aureus* (three of them detected by the BIOFIRE JI Panel in TBs), one *S*. *lugdunensis,* and one S*treptococcus* spp. (also detected by the BIOFIRE JI Panel and in the culture of TBs) (Table 2). Two detections observed in SF culture were missed by the BIOFIRE JI Panel SF, one *E. cloacae* and one *C. albicans*. In both cases, the BIOFIRE JI Panel detected these microorganisms in TBs.

Regarding the polymicrobial subjects (Table 5), the BIOFIRE JI Panel SF and TBs detected additional microorganisms as compared to SF and TB cultures. For the 19 polymicrobial subjects, 46 microorganisms were detected by the BIOFIRE JI Panel on SF and/or TBs, but missed by all cultures. Nine subjects’ SF and/or TB cultures yielded a single organism result when the BIOFIRE JI Panel SF and/or TB detected more than one organism. Notably, in three subjects, SF and TB cultures were negative, whereas at least one of the BIOFIRE JI Panel tests detected more than one organism.

## DISCUSSION

Microbiological culture is still the gold standard technique in osteoarticular infections. However, the sensitivity of culture is suboptimal, and approximately 10%–15% of patients have negative culture results despite having a true infection (3). For this reason, molecular approaches have been developed to increase the sensitivity of microbiological diagnosis, including specifically designed PCR kits for PJI (7).

Different clinical studies have demonstrated the potential of the BIOFIRE JI Panel to improve osteoarticular infection diagnosis (10–13). A multicenter evaluation documented its higher diagnostic precision compared to culture, shorter time to results for pathogen and resistance detection, and high sensitivity and specificity (8). These studies were performed using SF specimens, and this is the first study that evaluates its use in combination with SF and TBs (an off-label sample type) in two clinical settings.

Our results showed the potential application of the BIOFIRE JI Panel, enhancing the microbiological diagnosis of bone and joint infections, which can decisively influence the clinical management of patients. Our findings suggest that diagnostic yield is improved when employing the BIOFIRE JI Panel compared to conventional culture methods, particularly in cases of polymicrobial infections. Culture was used for comparison, given its widespread utilization across microbiological laboratories (14). For the monomicrobial infections analyzed in this study, good concordance between BIOFIRE JI Panel results and culture was observed for both SF (Kappa = 0.77) and TBs (Kappa = 0.80). The study performed by Lazic et al. (15) showed a moderate concordance (Kappa = 0.53) between SF cultures, tissue sample culture, and tissue sample multiplex PCR, using the Unyvero ITI cartridge, which is also designed for bone and joint infection. Another study, using the Unyvero ITI cartridge in tissue samples, showed an agreement of the cartridge results with culture results in about 82% of the cases (16). A strong concordance between PCR results from SF and PCR from TBs was also observed.

Furthermore, we have observed an increase in polymicrobial detections using the BIOFIRE JI Panel compared to culture methods, especially in tissue specimens. Previous studies have demonstrated that conventional culture techniques may overlook polymicrobial infections (17). These discrepancies may appear due to the overgrowth of one strain over more slowly growing microorganisms on agar plates and can also be influenced by prior antibiotic treatment. Additionally, we have observed positive BIOFIRE JI Panel results in TBs despite negative results in SF, and vice versa. This is partially explained by the fact that there were usually multiple tissue samples collected from the same subject, which may help increase diagnostic yield, especially for composite results presented. Furthermore, higher bacterial load in tissue samples that are attached to the implant compared to SF, particularly in chronic infection, may contribute to these findings.

The main limitation of our study is the lack of detailed clinical characteristics of the subjects. It was not possible to determine whether cases with culture-negative specimens but BIOFIRE JI Panel positive results were conclusively true positive or false positive by comparing to a clinician’s diagnosis. Another limitation is the lack of homogeneity in the number of TBs collected from each subject, where diagnostic yield is expected to be higher in subjects with more TBs tested. Additionally, like all syndromic PCR panels, not all microorganisms causing bone and joint infections are included. Therefore, this approach poses the risk of missed detections for microorganisms not targeted by the PCR panel. This technique should always be used alongside conventional culture methods to isolate microorganisms either not detected or not included in the panel and for antibiotic susceptibility testing. Moreover, in this study, only *Candida* species have been represented in the fungi; other molds relevant (but uncommon) to JI are not included. Results of the test should be evaluated according to the clinical context of the patient in multidisciplinary teams to optimize surgical revision strategy, antimicrobial stewardship, and improve patient management. It would be desirable to evaluate the usefulness of the BIOFIRE JI Panel on TBs in larger prospective studies.

In our study, we demonstrated that the BIOFIRE JI Panel improves diagnostic yield compared to conventional culture techniques. According to our results, a positive BIOFIRE JI Panel result from SF specimens had a higher diagnostic yield than culture in detecting the on-panel potential etiological agent.

It is important to note that the TB sample type is not an indicated sample type according to the BIOFIRE JI Panel’s intended use, and the performance of this sample type has not been evaluated by the manufacturer. While testing TBs on the BIOFIRE JI Panel is off-label, our analyses demonstrate that composite TB results on the BIOFIRE JI Panel were consistent compared to BIOFIRE JI Panel SF results and had a higher diagnostic yield compared to composite TB culture results.

If there is a high clinical suspicion of infection, and a PCR assay performed on the SF is negative or unfeasible, a PCR assay on TBs could be beneficial, particularly when a prompt result is needed for appropriate patient management. This approach may also be necessary when a PCR assay and culture from SF yield negative results, a TB culture also yields a negative result, yet there remains a strong suspicion of septic arthritis or prosthetic joint infection.

## SUPPLEMENTAL MATERIAL

File S1 spectrum.03402-25-s0001.xlsx Synovial fluid and tissue biopsy results, including PCR and culture results for each subject.

ASM does not own the copyrights to Supplemental Material that may be linked to, or accessed through, an article. The authors have granted ASM a non-exclusive, world-wide license to publish the Supplemental Material files. Please contact the corresponding author directly for reuse.
